# Ectoparasitosis, a rare cause of severe iron deficiency anemia: A case report

**DOI:** 10.1016/j.amsu.2021.102784

**Published:** 2021-09-06

**Authors:** Nuzhat Batool, David Song, Jonathan Vincent M. Reyes, Saad Ahmad, Arigirios Skulkidis, Talal Almas, Tarek Khedro, Maritza Brown

**Affiliations:** a Department of Internal Medicine, Icahn School of Medicine at Mount Sinai Hospital (Elmhurst Hospital Center), USA; b Royal College of Surgeons in Ireland, Dublin, Ireland

**Keywords:** Iron deficiency anemia, Ectoparasitosis, Pediculosis, Lice

## Abstract

Pediculosis is a common condition caused by an infestation of head and body louse (ectoparasites) and remains a public health concern. Generally, infestation presents as pruritus in children and has a benign course, but there have been a handful of cases reported in the literature describing severe iron deficiency anemia (IDA) in high-risk groups such as children, history of psychiatric disorder including depression, and low socioeconomic status. Though an uncommonly encountered etiology of anemia, the aim of this case is to increase awareness of a rare cause of severe anemia from an ectoparasites affecting a high risk population, even in developed countries.

## Highlights

• Pediculosis is a common condition caused by an infestation of head and body louse (ectoparasites). Infestation can be asymptomatic, or involve tingling sensations of the skin, irritability, insomnia, and pruritus.
• In severe cases of infestation, it can, on a rare occasion, manifest as severe iron deficiency anemia.
• The clinical course can vary from benign skin infestation to severe debilitating anemia, but with use of pediculicide and hygiene maintenance the condition can be easily eradicated.

## 1 Introduction

Pediculosis is a common condition caused by an infestation of head and body louse [1,2]. Lice are obligate parasites that are categorized into two main groups: Mallophaga: the chewing lice, and Anoplura: the sucking lice [3]. Mallophaga feed on skin debris, hair fragments, and sebaceous secretions found on the skin surface of both birds and mammals; while, Anoplura feed exclusively on mammals by piercing the skin of their host with sensitive retractable stylets designed to ingest the host's blood [3].

There are three types of lice that live on humans including *pediculus humanus capitis, pediculus humanus corporis and pthirus pubis* [4]. Transmission most commonly occurs by direct head to head contact with an infected individual, exchanging fully grown nymphs or unhatched nits; whereas, indirect transmission is less common but involves the use of contaminated clothing, sheets, hats, towels, and combs recently exposed by an infected person [5]. Infestation can be asymptomatic, or involve tingling sensations of the skin, irritability, insomnia, and pruritus [6]. Pruritus is driven by an antigenic response to salivary secretions of the Anoplura which serve as an anticoagulant during the feeding process [3]. Complications of excessive scratching include excoriations, boils, lymphadenopathy, and even alopecia [7]. Some cases may lead to secondary skin and soft tissue infections as a result of excoriations. However, in rare severe cases, it can manifest as iron deficiency anemia (IDA) without any underlying evidence of bleeding. The goal of this case is to highlight a rare complication of IDA and the management of pediculosis-associated anemia.

## 2 Case presentation

A 32-year-old undomiciled male with a past psychiatric history significant for schizophrenia, not on any medication and no other known medical history was brought into the emergency department after being found to be ill appearing in the street with disorganized behavior. On a physical exam, the patient appeared unkempt, with poor hygiene and innumerable lice grossly visible on his head, body, and clothing. There were diffuse excoriations on the abdomen, back, and upper and lower extremities. The vital signs were otherwise unremarkable. He was alert and oriented to self only. His thought content was tangential and irrational. Moreover, the patient did not seem distressed by the extensive presence of lice suggestive of chronic manifestation. Review of systems were negative for fevers, chills, night sweats, gastrointestinal symptoms, hematemesis, hemoptysis, hematochezia, melena, or hematuria and these symptoms did not resurface while he was admitted. He did not exhibit shortness of breath during initial evaluation and was seen pacing around the examination room, frequently rising up from the stretcher without complications.

Initial labs were significant for hemoglobin of 6.3 g/dL (normal: 13.5–17.5g/dL for male) with unknown baseline and eosinophilia of 16.5%. Further anemia workup revealed iron of 12 μg/mL (normal: 90–170 μg/mL), ferritin of 25 ng/dL (normal: 24–336 ng/dL), total iron binding capacity of 238 μg/dL (normal: 240–450 ng/dL), and normal haptoglobin 166. Hemoglobin electrophoresis was normal with HbA of 97.9%. Mean corpuscular volume was 68 fL and 3 million red blood cells per microliter with Mentzer index of 22 (Mentzer index >13 is suggestive of IDA; <13 is suggestive of Thalassemia). With the combination of Mentzer index and anemia work up, the patient was found to have IDA. Rest of his lab, including the complete metabolic panel, urine drug screen, coagulation panel, peripheral smear, hepatitis panel and human immunodeficiency virus, were unremarkable.

During the hospitalization, the patient received permethrin for lice eradication and was educated regarding the role of proper hygiene in preventing recurrence. The patient was subsequently transfused a total of two units of packed red blood cells as hemoglobin levels continued to drop down to 5.6 g/dL without evidence of bleeding and responded appropriately with improvement of hemoglobin to 8 g/dL post transfusion. At this time, the patient's vital signs continued to be stable with no signs of tachycardia or hypotension. Patient's digital rectal exam was performed revealing absence of blood. The gastrointestinal service was consulted for further evaluation of organic etiology of iron deficiency anemia and decision was made to pursue outpatient follow up for endoscopy/colonoscopy which later revealed internal hemorrhoids without masses or signs of bleedings. Patient was diagnosed with IDA and started on a ferrous sulfate 325mg tablet daily for management. The Patient had subsequent follow up which revealed improvement in his overall hygiene with eradication of his lice and stable hemoglobin in the 8 g/dL as a new baseline.

## 3 Discussion

The diagnosis of pediculosis requires the direct visualization with the naked eye or with assistance of a wood lamp to confirm the presence of live adult lice or nymphs as nits alone are insufficient for diagnosis as they may persist for weeks to months after successful treatment [8]. Pediculosis commonly has a relatively benign course; however, there are a few cases in the literature describing severe infestation causing iron deficiency anemia [[9], [10], [11], [12]]. The exact mechanism of this process is not well understood, but in theory, severe infestations can cause blood loss of about 21ml per month which then manifests as anemia from chronic blood loss in appropriate settings [13].

The populations at high risk for iron deficiency anemia in the setting of pediculosis are children, patients with a history of psychiatric disorder, including depression, and low socioeconomic status [2,9,11]. In order to establish a diagnosis, other causes of iron deficiency anemia must be ruled out. Common lab tests conducted include peripheral smear, iron panel, coagulation panel, and hemoglobin electrophoresis. Some patients also underwent esophagoduodenoscopy, colonoscopy, and computed tomography of abdomen and pelvis based on clinical suspicion of the source of bleed [11].

Three different therapeutic regimens are used to eliminate head lice: therapeutic wet combing, topical application of pediculicide (Permethrin 1%), and oral treatment with Ivermectin which is used in refractory cases [14,15]. As opposed to head lice, body lice often doesn't require pediculicide treatment; instead, simple showering and laundering clothing in hot water is the first line of treatment [8]. With the treatment of infestation, over time, body iron stores and hemoglobin recovers. In some instances, ferrous sulfate can be supplemented for IDA just like in our patient with strict follow-up.

## 4 Conclusion

Ectoparasites generally infest the head and body. It is primarily asymptomatic; however, it can cause IDA on rare occasions, even in developed countries. This case is presented to increase awareness, and the diagnosis and management of IDA from ectoparasite. The clinical course can vary from benign skin infestation to severe debilitating anemia, but with use of pediculicide and hygiene maintenance the condition can be easily eradicated.

## Conflicts of interest

None.

## Sources of funding

None.

## Ethical approval

Obtained.

## Consent

Obtained.

## Author contribution

NB wrote the abstract, case, study concept, design, conclusion; DS, JVR, AS, SA reviewed paper, wrote discussion, DS, TA, MB performed final edits.

## Registration of research studies

1. Name of the registry: NA
2. Unique Identifying number or registration ID: NA
3. Hyperlink to your specific registration (must be publicly accessible and will be checked): NA

## Guarantor

Talal Almas, RCSI University of Medicine and Health Sciences, 123 St. Stephen's Green Dublin 2, Ireland, Talalamas.almas@gmail.com, +353834212442.

## Declaration of competing interest

None.

## Disclosure

None.
